# COSMETIC CAMOUFLAGE IN VITILIGO

**DOI:** 10.4103/0019-5154.70663

**Published:** 2010

**Authors:** K N Sarveswari

**Affiliations:** *From the Consultant Dermatologist, Sundaram Medical Foundation, Dr. Rangarajan Memorial Hospital, Chennai, Tamil Nadu, India*

**Keywords:** *Vitiligo*, *dermatology life quality index*, *camouflage*

## Abstract

Vitiligo is not a life–threatening nor a contagious disease. But the disfigurement of vitiligo can be devastating to its sufferers, especially dark-skinned individuals. Available treatment options are disappointing and sufferers often use various forms of camouflage. Remedial cosmetic cover creams help conceal the blemish of vitiligo at least temporarily. A high concentration of pigment is incorporated into water–free or anhydrous foundations to give a color that matches the patient’s skin, thereby concealing vitiligo patches. The article highlights the content and technique of application of these creams.

## Introduction

Camouflage is derived from the French word, ‘Camoufler’ which means ‘to blind’ or ‘veil’. Also known as protective concealment, it means to disguise an object in plain view, for the purpose of concealing it from something or someone. In mammals, the color of skin/fur plays an important role in camouflage and prey-predator relationship. That is why early *Homosapiens* living naked in the depth of the yesteryear African forest was black-skinned.

A great example of this is the ‘stick insect’, which becomes almost invisible due to the shape of its body, coloration and slow movement. It looks and acts like a twig on a bush or tree.

The art of camouflage has been well exploited by the army to hide from the enemy. To make their face match a jungle backdrop, soldiers have used camouflage cosmetics that come in rich greens and browns. The soldiers paint their faces in blotchy patterns to match their surroundings.

Another camouflage cosmetic was pioneered during the Second World War to help pilots who were badly burnt by making use of plastic surgery and camouflage cream. These creams did not camouflage the person but were used to conceal blemishes on the skin so that they were not obvious.[1]

During the 1950s, Joyce Allsworth researched and implemented the concept of remedial skin camouflage within the UK. She went on to form the British Association of Skin Camouflage (BASC) in 1985.[2]

The BASC defined remedial cosmetic skin camouflage as being the ‘the art of concealing a discoloration, blemish or scar with the application of specialist camouflage creams that are matched to the surrounding skin tone.’

Nowadays, it is used to conceal abnormalities from various dermatological disorders, especially vitiligo.

Vitiligo is not a life–threatening or a contagious disease, but, its disfiguring signature often devastates the sufferers, specifically the dark-skinned type. For many, it is not just a cosmetic problem but a major social dysfunction that seriously curtail their ability to lead normal, professional, social or married lives.

The results of standard and experimental treatment options for vitiligo are disappointing with only partial results. In addition, most require treatment periods, lasting months or years, before re-pigmentation occur. Sufferers use practices of concealment such as the use of camouflage make-up, clothing, hair dye and specific bodily movements (like putting hands in their pockets).

On asking vitiligo patients whether they made a conscious effort to hide or camouflage their discoloration, 47% said that they did attempt to cover while 29% said they did so sometimes. Most (87%) female patients, especially the dark-skinned type, attempted to camouflage vitiligo all the time or sometimes.[3]

In a cohort study of vitiligo patients, Ongenae *et al*. showed that the Dermatology Life Quality Index (DLQI) improved after the use of camouflage and hence, recommended the use of camouflage in patients with higher DLQI scores.[4]

Camouflage improves the appearance of the individual, as the focus is no longer on the discoloration that the person wishes to hide, thereby improving their confidence and self-esteem.[5]

Camouflage may be permanent or temporary.

Permanent camouflage is obtained with a cosmetic tattoo. Unlike pigments used for ritual or symbolic tattoos, cosmetic tattoos are inert iron oxides that are available in more than 15 shades.[5]

Various tattoo pigments include:

- White: Titanium dioxide
- Red: Cinnabar, Mercuric sulphate
- Black: Iron oxide
- Yellow: Cadmium sulphate
- Camel yellow: Iron oxide
- Light brown: Iron oxide
- Dark brown-: Iron oxide
- All tattoo pigments must be autoclaved before use.

**Figure 1:**
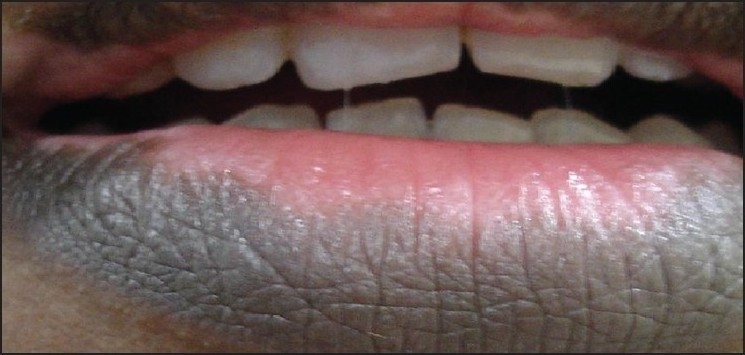
Vitiligo on lips (Photograph courtesy Dr. G. Ravichandran)

**Figure 2:**
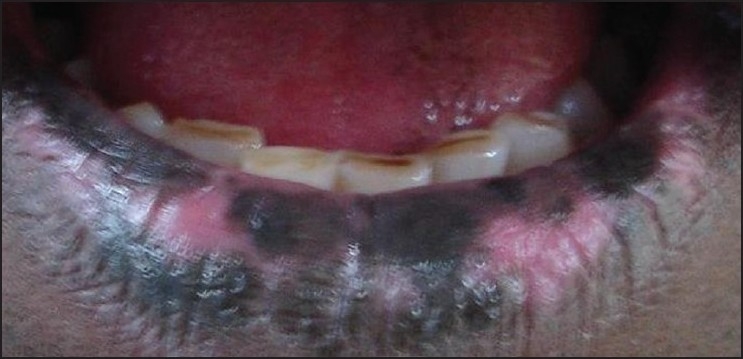
Vitiligo on lips after tattooing (Photograph courtesy Dr. G. Ravichandran)

The color is implanted into the dermal layer with specialized techniques and cannot be washed off [Figure 1] Very satisfactory results are obtained when only small areas of the face, [Figure 2] particularly in the perioral area, and the dorsal hands are involved. Dark photo types are more easily treated than people with fair skin.[6]

Cosmetic results are strongly dependent on the doctor’s or the technician’s skill in perfectly matching the color of the tattoo with the color of the surrounding skin area. The colors of the tattoo fade naturally over time, requiring periodic maintenance, usually 2–5 years.

However, unless performed under universal precautions with scrupulous sterile technique including sonic cleaning and autoclaving of the instruments, tattooing carries a risk of transmitting infectious diseases, including viral hepatitis and human immunodeficiency virus (HIV) infection.

Granulomatous foreign bodies, allergic, and photo allergic reactions have been reported from tattooing. Mercury accounted for most of the allergic reactions to red tattoos, but its restriction in the USA has ameliorated this allergy.

## Temporary Camouflage

Temporary camouflages include:

1. Liquid dyes
2. Indigenous preparations
3. Remedial cosmetic camouflage
4. Self-tanning products

### Liquid dyes

Potassium permanganate, indigo carmine, bismarck brown, and henna paste are commonly used to camouflage vitiligo. These are used to provide an immediate, natural, amber-like shade that can be adjusted to the desired shade by applying a single layer for a lighter shade or an additional layer to get a darker color. These are washed away easily.

### Indigenous preparations

Loha Bhasma: Exposing iron filings to sunlight with a liberal lacing of vegetable extracts yields a powder that was commonly used for anemia. This provided a good coverage for vitiligo but the disadvantage was that it rubbed off easily.[7]

Swarna Karani: Sugathan *et al*. reported that a swarna karani, a type of clay mixed with oils and henna was an effective camouflage in vitiligo.[7]

None of these give acceptable match for pigmented skin.

### Remedial cosmetic camouflage

The uniform application of thin films of selected opaque cosmetics with light-reflecting ingredients is very effective for covering or at least reducing the visual impact of white patches. Products for covering vitiligo are specific and quite different from other common cosmetic make-ups.

There are four basic facial foundations:[8]

1. Oil-based foundations these are water-in-oil emulsions containing pigments suspended in oil, such as mineral oil or lanolin alcohol. Sometimes, vegetable oils (coconut, sesame, safflower) and synthetic esters are also used.
  The water evaporates from the foundation after application, leaving behind the pigment in oil on the face. This creates a moist feeling and is especially used for patients with dry skin.
2. Water-based facial foundations are oil-in-water emulsions containing a small amount of pigment in which the pigment is emulsified with a relatively large quantity of water. These are appropriate for minimally dry to normal skin. They are usually packaged in a bottle.
3. Oil-free facial foundations contain no animal, vegetable, or mineral oils. They contain other oily substances such as dimethicone or cyclomethicone. These are designed for oily-skinned individuals because they leave the skin with a dry feeling. Silicon is noncomedogenic, hypoallergenic and thus, is tremendously popular. These are usually liquids packaged in a bottle.
4. Water-free or anhydrous, foundations are waterproof. Vegetable oil, mineral oil, lanoline, alcohol and synthetic esters form the oil phase that may be mixed with waxes to form a cream. High concentrations of pigment can be incorporated into the formulation, yielding an opaque facial foundation. The coloring agents are based on titanium dioxide with iron oxides, occasionally in combination with ultramarine blue. Titanium dioxide acts as a facial concealing or covering agent. These products can be dipped from a compact or stroked from a stick. These foundations are well suited for use with patients who require facial camouflaging.

There are three basic approaches to basic camouflage cosmetics, *i.e*. contour correction, color correction, or a combination of both.

Color abnormality camouflaging: Pigmentation defects can be camouflaged by applying an opaque cosmetic that allows none of the abnormal underlying skin tones to be appreciated. The most effective way to hide irregular coloring would be to apply a camouflage cosmetic of the complementary color; this will neutralize the underlying unwanted color. For instance, if we want to hide a purple tattoo, an orange camouflage must be applied first, followed by the application of a cream camouflage cosmetic that matches the skin. Skin areas that are lighter or darker than desired can be camouflaged by applying facial foundations with the appropriate amount of brown pigment to hide the defect. Table 1 summarizes these color-blending techniques.

**Table 1:** Color-blending techniques

Facial color	Disease process	Foundation color
Red	Psoriasis, lupus, rosacea	Green undercover foundation
Yellow	Solar elastosis, dialysis	Purple undercover foundation
Brown Pigmentation	Cholasma, lentigenes	White undercover foundation
Hypopigmentation and depigmentation	Postinflammatory, vitiligo	Brown undercover foundation

Finally, facial powders that match the skin tone may be applied to set the camouflage makeup and prevent it from smudging off. They are valuable cosmetics that provide coverage of complexion imperfections, oil control, a matte finish, and increased tactile smoothness to the skin. They predominantly contain talc known as hydrated magnesium silicate and high amounts of covering pigments. Iron oxide is the main pigment but other inorganic pigments such as ultramarine; chrome oxide, and chrome hydrate may also be used. These powders are designed to augment the underlying skin and foundation tones.

### Technique of application

**Figure 3:**
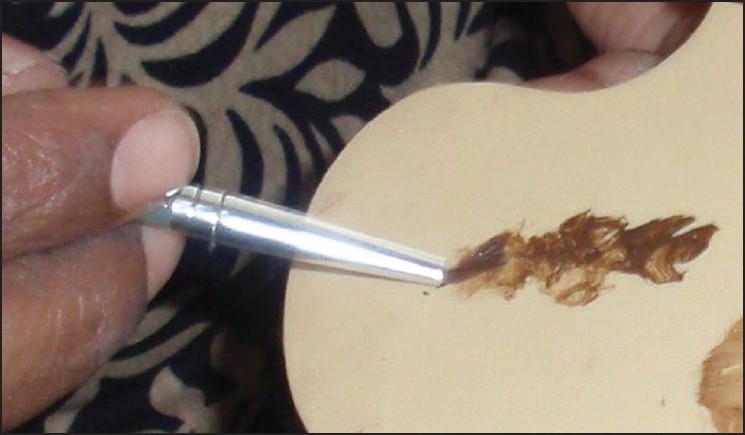
Blending of colors to match skin tone

**Figure 4:**
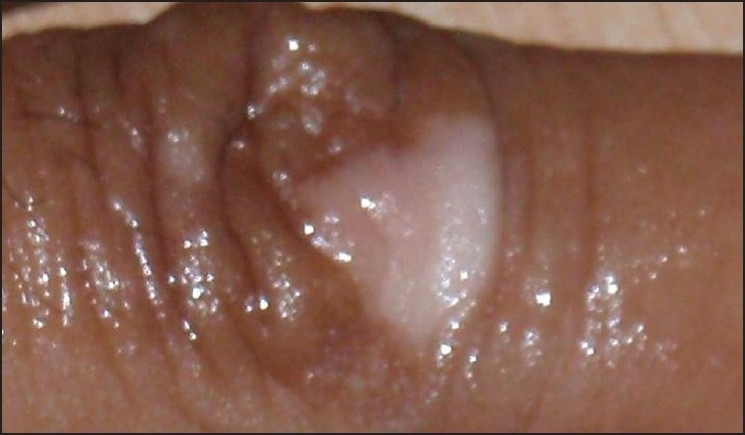
Vitiligo on hands

**Figure 5:**
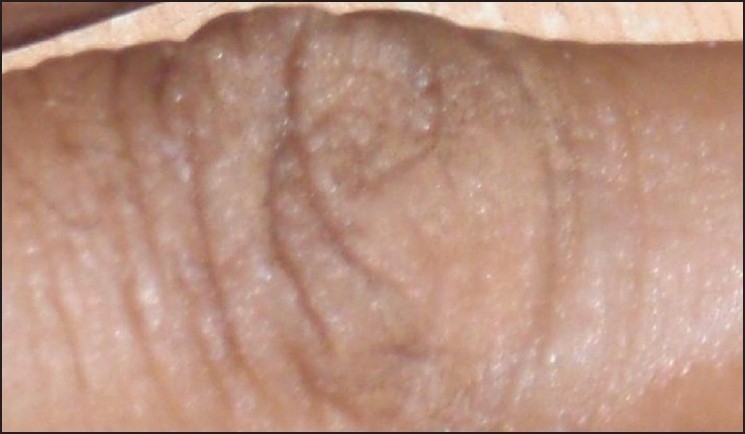
Vitiligo on hands after camouflage cover creams

1. Cleanse the skin.
2. Choose a make-up base closest to the patient’s natural skin color.
3. Blend with other colors so as to match the patient’s skin tone. No more than three colors should be combined. The underlying vitiligo is considered one color and is usually yellow in sallow skin and red if the patient is darker [Figure 3].
4. Blending is accomplished by applying a small amount of the make-up to the back of the hand.
5. Apply cream sparingly from the center (thin coat) of blemish and blend into normal skin [Figures 4 and 5].
6. Wait for 5 min for setting of cream.
7. Translucent loose powder must be pressed on top of the foundation with cotton wool.
8. Multiple thin layers may be used for maximum coverage.
9. Takes one hour for color to settle and become waterproof, nongreasy, and resistant to sunlight and smudging so that it will not rub off on clothing.
10. Oily cleanser is used to remove the camouflage at night.
11. Soap and water cleansing may be done after this.
12. Moisturizer may be applied.

Self-tanning products contain dihydroxyacetone 3–5% (DHA) which is a sugar that binds with the amino acids of the corneum layer, inducing the production of colored components that change yellow to brown, giving the skin a tanned effect. DHA dyes are easy to apply—they are neither dirty nor greasy. The pigmentation appears a few hours later and the application has to be repeated until the desired result is obtained, and more times in a week, because DHA- induced pigment reduces with normal exfoliation of the epidermis. These dyes do not provide protection against sunburn and psoralen phototherapy can be used.

Rajatanavin *et al*.[10] found that color matching was achieved by using a higher concentration of DHA in darker-skinned subjects. Most of the vitiligo patients (88.9%) reported moderate to marked satisfaction with the cosmetic results of 6% DHA cream.

## Conclusion

The skin, like a cloak that covers us all over, is the oldest and the most sensitive of our organs, our first medium of communication, and our most efficient of protectors. Despite all these unique factors, it cannot offer protection from the social ostracism and psychological crippling that a disease like vitiligo can cause.

Vitiligo often affects the minds of patients, lowering their confidence and self-esteem and affects their relationship with family and friends.

Skin camouflage represents a quick, noninvasive treatment, which by disguising vitiligo patches, helps to significantly reduce the patient’s distress. Whether the patient uses it everyday allowing them to work and enjoy normal social activities without being self-conscious, or just uses it for special occasions; camouflage can make all the difference to the patient’s quality of life.
